# Using injectable Platelet-Rich fibrin to improve recovery after impacted lower third molar extraction: a randomized controlled clinical trial

**DOI:** 10.1007/s00784-025-06563-3

**Published:** 2025-09-19

**Authors:** Angelo Aliberti, Mauro Mariniello, Marco Bergaminelli, Pasquale Dolce, Dario Gargiulo, Gilberto Sammartino, Gianrico Spagnuolo, Roberta Gasparro

**Affiliations:** 1 Department of Neurosciences, Reproductive and Odontostomatological Sciences, University of Naples Federico II, Via Sergio Pansini 5, Naples, 80131 Italy; 2 Dipartimento di Salute Mentale, Odontoiatria per Bisogni Speciali, ASL Salerno, Salerno, Italy; 3 Department of Translational Medical Sciences, University of Naples Federico II, Via Pansini 5, Naples, 80131 Italy; 4 Therapeutic Dentistry Department, Institute of Dentistry, Sechenov University, Moscow, 119991 Russia

**Keywords:** Impacted third molar, Swelling, Pain, Wound healing, Injectable platelet rich fibrin

## Abstract

**Objectives:**

The aim of this RCT was to clinically evaluate swelling, pain and wound healing following submucosal infiltration of injectable Platelet-Rich Fibrin (i-PRF) after extraction of impacted lower third molar.

**Materials and methods:**

The study was designed as a double-blinded, parallel group, randomized controlled clinical trial. 56 patients were divided into 2 groups: in the control group the socket was left to heal spontaneously, while the test group was treated with submucosal infiltration of i-PRF. Swelling was measured with a flexible ruler using the diagonals joining Trago and Pogonion, Gonion and labial cleft. The swelling was calculated as the sum of these diagonals before surgery, on day 3 and 7. Pain was assessed by VAS scale until the 7th postoperative day. Surgical wound healing was evaluated by the Healing Index by Landry on 3, 7 ,14 and 21st day postosperatively. The relation between duration of intervention and total swelling was also evaluated.

**Results:**

56 patients were enrolled in this study (28 for the test group and 28 for the control group). Randomization resulted in groups with similar baseline characteristics. No patients were lost during the follow-up and no adverse events were noted. On day 3 the total swelling was 12.7 ± 0.92 mm for control group and 12.1 ± 0.75 mm for test group; on day 7 it was 12.3 ± 0.88 mm for control group and 11.7 ± 0.73 mm for test group. A statistically significant difference was found on day 3 (*p* = 0.006) and on day 7 (*p* = 0.018). The relation between total swelling and duration of intervention was not statistically significant (*p* = 0.276). A significant reduction in pain scores was observed in the i-PRF group on days 1 and 3 (*p* < 0.001). Surgical wound healing also showed statistically significant improvement in the i-PRF group at all time points (days 3, 7, 14, and 21; *p* < 0.05).

**Conclusions:**

Within the limit of our study, this randomized controlled clinical trial suggests that submucosal infiltration of i-PRF after the extraction of impacted lower third molars effectively reduces postoperative swelling and pain, while also promoting faster wound healing.

**Clinical Relevance:**

The adjunctive use of injectable platelet-rich fibrin (i‑PRF) after surgical removal of impacted mandibular third molars may contribute to improve early postoperative outcomes by attenuating soft tissue inflammation, reducing pain intensity, and accelerating wound healing. By potentially lowering the need for nonsteroidal anti-inflammatory drugs (NSAIDs) and minimizing associated adverse effects, i‑PRF represents a minimally invasive, autologous approach that could enhance patient comfort, reduce recovery time, and support faster return to daily activities.

**Supplementary Information:**

The online version contains supplementary material available at 10.1007/s00784-025-06563-3.

## Introduction

The surgical extraction of mandibular third molars is among the most common oral surgical procedures, with an impaction prevalence ranging from 33 to 59% [1–3]. Impacted third molars are associated with various complications, including pericoronitis, trismus, odontogenic infections, distal caries of the adjacent second molar, cysts, and even tumors [4, 5]. These conditions increase patient morbidity, prolong recovery, and impose additional healthcare costs. Postoperative sequelae, particularly pain, swelling, and trismus, are common due to the acute inflammatory response following surgical trauma [6–8]. Reducing these sequelae is crucial for the success of surgical procedures [9]– [10]. Postoperative swelling occurs due to tissue damage, elevation of muscle attachments, and direct damage to blood and lymphatic vessels during surgical extraction [11]. On the other hand, surgical extraction of impacted wisdom teeth usually requires the removal of a large amount of bone to expose the impacted tooth.

Conventional strategies to minimize these complications include a variety of pharmacological and non-pharmacological methods as nonsteroidal anti-inflammatory drugs (NSAIDs) [12], administration of dexamethasone and methylprednisolone, laser therapy [13, 14], hyaluronic acid spray [15] or using piezoelectric bone surgery [16]. Corticosteroids are among the most commonly approaches to reduce postoperative symptoms, minimize swelling and limit the mouth opening (trismus) following third molar extractions. However, they are associated with potential adverse effects, including delayed wound healing and increased susceptibility to infection [17, 18].

Autologous platelet concentrates have emerged as biologically active adjuncts that promote soft tissue repair and modulate the inflammatory response by releasing cytokines and growth factors. Platelets release key growth factors that promote tissue repair and angiogenesis, including PDGF (platelet-derived growth factor), VEGF (vascular endothelial growth factor), and TGFβ1 and β2 (transforming growth factor β1 and β2), which can stimulate cell proliferation and increase angiogenesis [19, 20]. Various techniques have been developed for obtaining autologous platelet concentrates.

PRF, first developed by Choukroun et al. [21], is a second-generation platelet concentrate produced using a simplified processing technique. Unlike other platelet concentrates, PRF does not dissolve quickly after use; it efficiently collects platelets and leukocytes and activates platelets in the process, resulting in significant embedding of platelet and leukocyte growth factors into the fibrin matrix. Many studies have shown that the application of PRF to periodontal defects, cysts, tooth extractions, and sinus floor augmentation can accelerate wound healing, stimulate bone and soft tissue regeneration, and reduce inflammation, pain, and adverse side effects [22–24].

While traditional PRF has demonstrated beneficial effects in promoting tissue healing and reducing inflammation, the advent of injectable platelet-rich fibrin (i-PRF) offers an even more versatile approach [25]. i-PRF is a liquid form of PRF that can be easily injected into surgical sites, allowing for precise delivery of growth factors directly to areas that require healing [26]. This formulation is believed to enhance both soft tissue regeneration and bone healing through the release of various growth factors such as vascular endothelial growth factor (VEGF), platelet-derived growth factor (PDGF), and transforming growth factor-beta (TGF-β) [27]. The use of i-PRF in oral surgery, particularly following the extraction of lower impacted third molars, has recently gained interest. Studies have suggested that i-PRF can play a crucial role in reducing the incidence of post-extraction complications, including dry socket, by accelerating tissue repair and reducing inflammation [28]. Furthermore, it has been shown to shorten healing times and promote better overall outcomes, leading to greater patient satisfaction [29],. Despite these promising findings, the literature remains inconsistent regarding the efficacy of iPRF in reducing postoperaƟve complications following third molar extractions. Therefore, this randomized controlled clinical trial aimed to assess the effects of submucosal i-PRF infiltration on postoperative swelling, pain, and surgical wound healing after mandibular third molar removal.

## Materials and methods

### Study design

The study was designed as a double-blinded, parallel group, randomized controlled clinical trial. The study was approved by the Ethics Committee of the University of Naples Federico II (protocol number: 418/20) and was registered on ClinicalTrials.gov (NCT060073535). The trial was conducted according to the CONSORT statement (http://www.consort-statement.org/).

56 patients (M = 31; F = 25, aged 18–51) who visited the Department of Oral Surgery for lower third molar extraction were recruited. Patient eligibility was assessed during the initial anamnesis stage at the time of medical record compilation.

###  Patients’ selection

Patients were included based on the following criteria:

- Male and female patients;
- Age > 18 years;
- Need for extraction of a fully or partially impacted lower third molar;
- Similar degree of difficulty in third molar extraction [22];
- Ability to understand and sign informed consent.
- ASA 1 patients.

Patients were excluded if they met any of the following conditions:

- Patients aged < 18 years;
- History of recent analgesic or anti-inflammatory use within 2 weeks before surgery;
- Presence of medical conditions contraindicating surgery;
- Pregnancy or lactation;
- Immunocompromised patients;
- Smoking patients;
- Alcohol and drug abuse;
- Lack of cooperation;
- Dysplastic complication affecting the tooth;
- Routine use of medications that may interfere with healing (e.g. bisphosphonates, corticosteroids).
- Failure to adhere to examinations for the duration of follow-up;
- Consumption of more than two doses of prescribed anti-inflammatory/analgesic drugs.

Participants were randomly assigned to one of two groups:

- Control group: natural healing without i-PRF injection (28 patients).
- Test group: i-PRF injection (28 patients).

**Fig. 1:**
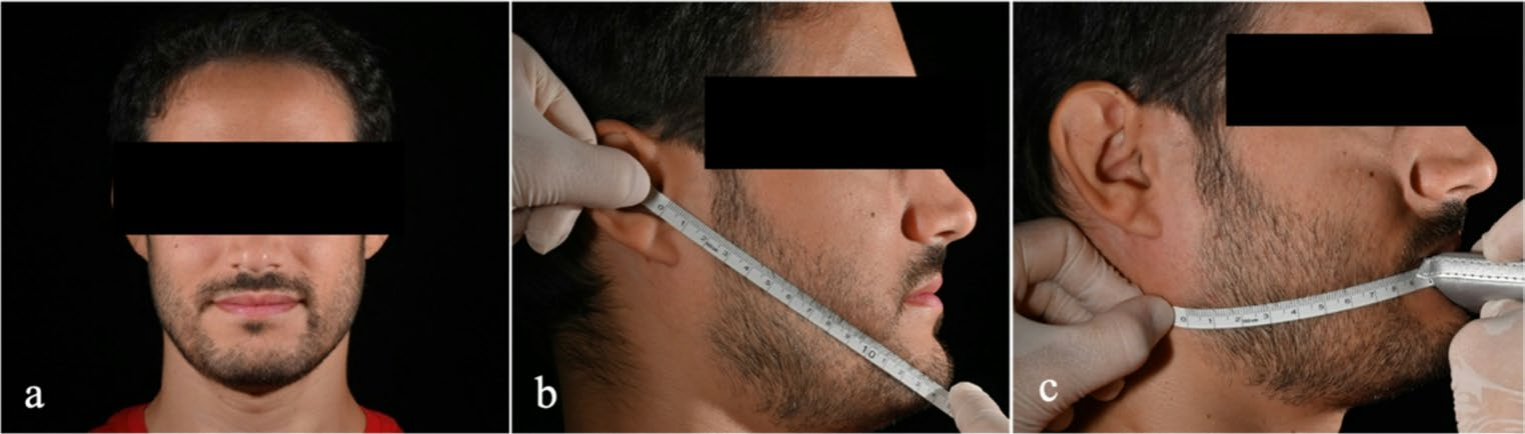
Photographic documentation of the clinical case: (**a**) extra-oral frontal photography; (**b**) extra-oral latero-lateral photograph of the affected emilate with a flexible ruler using the diagonal joining Trago and Pogonion; (**c**) extra-oral latero-lateral photograph of the affected emilate with a flexible ruler using the diagonal joining Gonion and labial cleft

All patients were informed about the nature of the study and were required to understand and sign the informed consent form. Before surgery, photographic documentation was obtained, including a frontal extraoral photograph and a lateral extraoral photograph of the affected hemi-mandible. (Fig. 1) A flexible ruler was used to measure diagonals connecting anatomical landmarks, including the tragus and pogonion, as well as the gonion and labial cleft.

### Randomization and blinding

The administration of i-PRF was randomly assigned to each patient. Randomization was conducted by a single examiner using a commercially available software (PASS, NCSS). The assigned numbers were sealed in identical opaque envelopes, which were drawn by an individual not involved in the study. Allocation to the test or control group was determined only after the extraction of the lower third molars by opening the envelope.

Patients were informed via written consent that they would be randomly assigned to one of the two groups and that they might undergo tooth extraction without the administration of platelet concentrate.

All treatment procedures were provided by one oral surgeon.

The principal investigator was not blinded due to the experimental procedures. However, the co-investigator responsible for measurements remained unaware of the specific procedure applied to each patient. The examiner and statistician were blinded. All patient data were securely stored in a locked location accessible only to the principal investigator and clinicians responsible for data collection. Patient identities were protected, known only to study participants, and processed by an experienced statistician to ensure confidentiality and data integrity.

### Surgical procedure and post-operative management

**Fig. 2:**
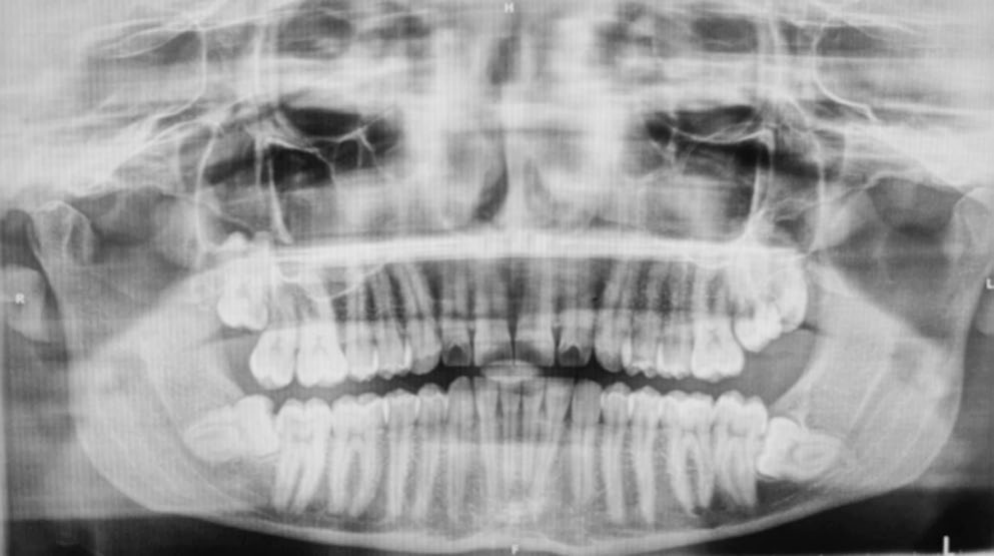
Pre-operative orthopantomography: osteomucosal inclusion of dental elements 38 and 48

All surgical procedures were performed under standardized conditions by the same experienced oral surgeon to ensure consistency. Preoperative radiographic evaluation was conducted using orthopantomography or cone-beam computed tomography (CBCT) when necessary. (Fig. 2). After conventional antibiotic therapy with Amoxicillin and Clavulanic Acid 1 g/12 h, from the day before surgery, local anesthesia with mepivacaine 2% with adrenaline 1:100,000 was performed.

**Fig. 3:**
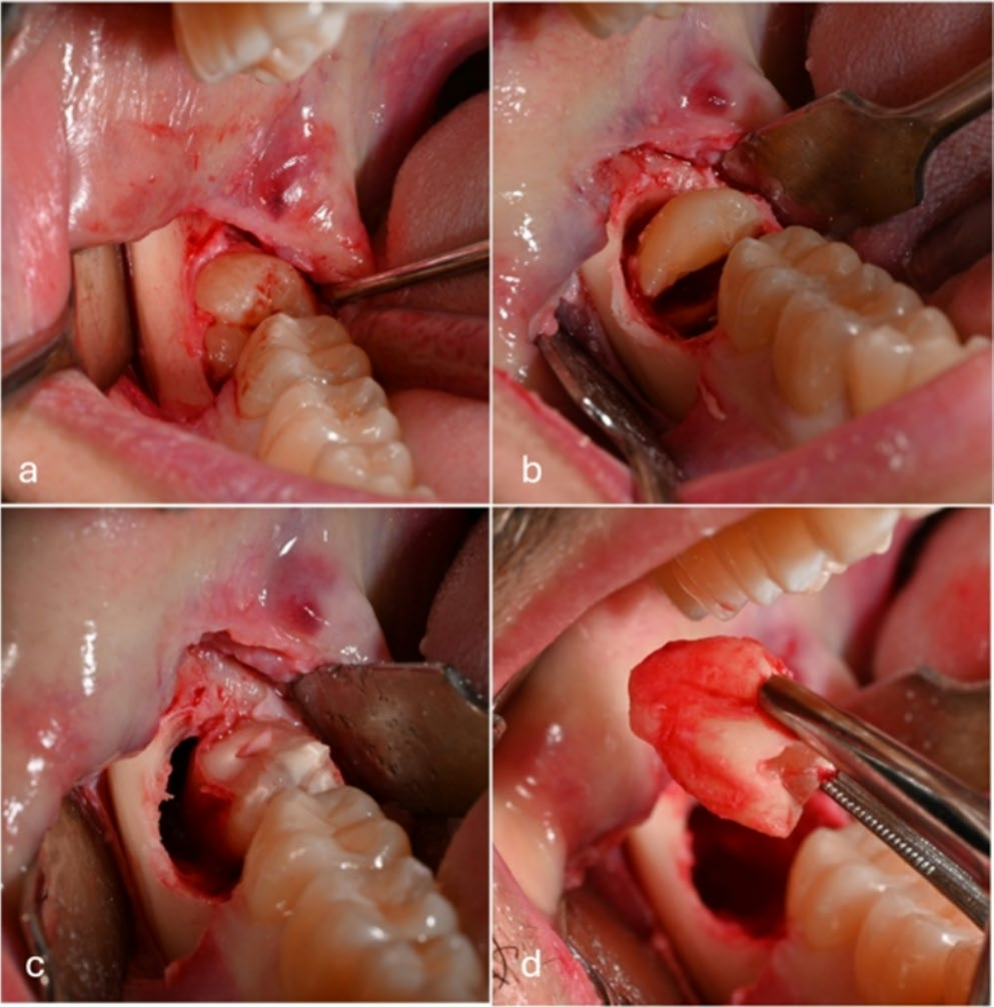
Operative procedure of dental avulsion of tooth 48 in osteomucosal inclusion: (**a**) Modified Archer’s flap and full-thickness tunnelling; (**b**) perimetral osteotomy and coronal odontotomy; (**c**) rhizectomy and dislocation: (**d**) root avulsion and residual alveolar cavity revision

A mucoperiosteal incision was made from the vestibular portion of the ridge to the erupted portion (partial inclusion) or to the distolingual angle of the second molar (total inclusion), continuing in the vestibular gingival contour of the second molar, extending into the fornix with a 45° release cut, avoiding involving the zenith of the interdental papilla between the second and first molars. The flap thus was full thickness elevated. If the tooth was totally inside the bone, it was necessary to expose the crown of the tooth by performing an osteotomy around its entire perimeter. To limit the trauma to the patient resulting from a large osteotomy, an odontotomy was performed to free the tooth from any undercut, then dislocation and avulsion were performed (Fig. 3). After extraction, hemostasis control was performed, and a 4/0 resorbable monofilament suture was applied. Immediately after the operation, the surgeon opened the envelope containing the patient’s group information and, for test group, performed the submucosal i-PRF infiltration.

Postoperative care was identical for all participants. Ibuprofen 400 mg was recommended only as needed for pain relief, with a maximum of two doses permitted during the follow-up period; patients requiring more than two doses were excluded from the analysis. All post-operative instructions (liquid and lukewarm diet, rest and daily oral hygiene) were given. Each patient was called back for follow-up and swelling measurements after 3 days and 7 days for photo documentation and swelling measurements and at 14 and 28 days to assess surgical wound healing. On day 7 the sutures were removed.

### Method of Preparation of i-PRF

**Fig. 4:**
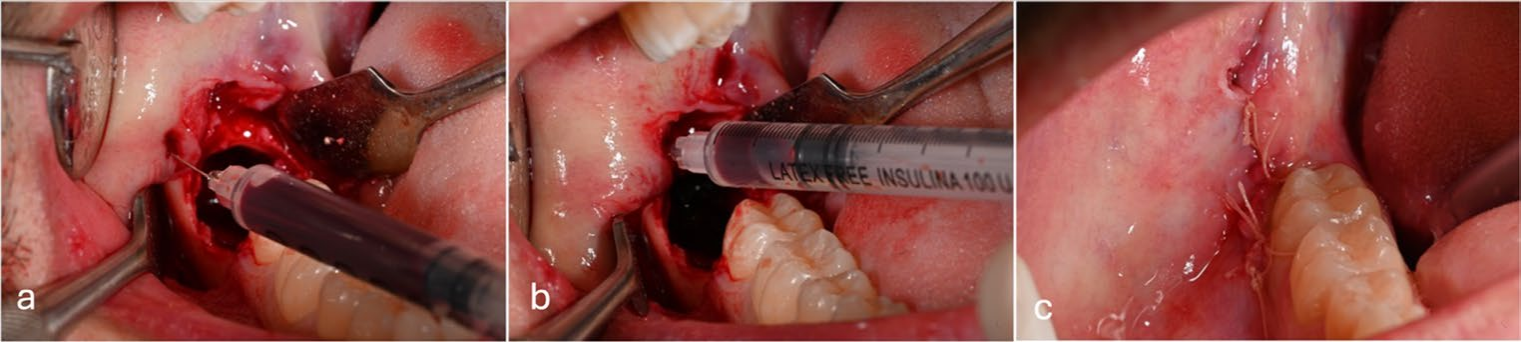
**a**-**b**): submucosal infiltration of i-PRF circumferentially around the surgical site; **c**) application of monofilament resorbable 4/0 suture with detached stitches

20 ml of venous blood was taken from the antecubital vein of all test patients and collected in 2 plastic tubes (Vacutainer, Becton& Dickinson, Rutherford, NJ) containing no anticoagulant or gelling agent. The tubes were placed in a centrifuge (DUO Centrifuge, Nice, France) at 700 rpm for 3 min, at the end of which the blood was separated into 2 fractions: the layer of i-PRF injectable liquid, more superficial, and the layer of red blood cells, in the deep part. From each tube, 5 mL of i‑PRF was aspirated from the upper layer using a sterile syringe. The time from the end of centrifugation to submucosal application was strictly standardized at < 2 min to ensure maximal preservation of bioactive components. (Fig. 4)

### Measurement of the outcomes

A researcher who did not participate in the surgical procedures and randomization, and blinded to the group allocations, performed the measurements of the outcomes: swelling, pain, time of intervention and healing of the surgical wound.

#### Swelling

Facial swelling was quantified by summing two standardized diagonal measurements: (1) from tragus to pogonion, and (2) from gonion to labial commissure [30]. Measurements were taken with a flexible ruler were recorded preoperatively (baseline) and on postoperative days 3 and 7. To improve reproducibility, each measurement was taken three times consecutively, and the mean value was used for analysis.

#### Pain

Pain was assessed using a Visual Analogue Scale (VAS), where 0 indicated no pain and 10 represented the worst pain imaginable. The VAS is a validated and reliable tool for assessing subjective pain in oral surgery, demonstrating high test–retest reliability and sensitivity to clinical changes. Patients were instructed to record their pain levels daily, starting from the first day post-surgery until postoperative day 7.

#### Duration of intervention

The duration of the intervention was counted from incision until tooth removal and was evaluated in minutes.

#### Healing of the surgical wound

Surgical wound healing was evaluated by the Healing Index (HI) introduced by Landry et al. [31] This index evaluates the parameters of tissue color, bleeding response to palpation, presence of granulation tissue, characteristics of the incision margins, and the presence of suppuration. This index assesses wound healing using scores from 1 to 5: a wound with very poor healing receives a score of 1, whereas excellent healing receives a score of 5. The index has been widely used in periodontal and oral surgery research and is considered a clinically valid indicator of soft tissue healing progress.

### Statistical analysis

A priori sample size calculation was conducted to determine the number of participants required to detect a significant difference in the primary endpoint (swelling). Based on a previous study [15] which reported a swelling mean of 12.8 ± 0.83 mm in one group and 13.36 ± 0.56 mm in the other group, we calculated that a sample size of 56 participants (28 per group) would be needed to detect this difference with a two-sided significance level of 0.05 and 80% power.

Continuous variables were presented as mean ± standard deviation (SD) or median with interquartile range (IQR), depending on data distribution, while categorical vari-ables were expressed as absolute and relative frequencies (%).

Baseline characteristics were reported separately for each group and assessed for clinically meaningful imbalances.

To evaluate longitudinal changes in swelling, VAS and wound healing, Linear Mixed Effect model or Linear Quantile Mixed Effect Model (LQMM) were applied. The model included random intercepts for subjects to account for the repeated measurements over time. Fixed effects included the time variable and the group variable to assess differences between experimental and control groups. The interaction between time and group was tested to determine if the change over time varied by group. For the LQMM, the quantile level was fixed at the median and p-values were computed through boot-strapping. Post-hoc pairwise comparisons were adjusted using the Tukey method.

A linear regression model was used to evaluate the relation between duration of intervention and total swelling at day 3, controlling for treatment group.Statistical analysis was conducted in R statistical software (version 4.3.3), with statistical significance set at α = 0.05 for all tests. The lme4 package was used Mixed-effects modeling, while the lqmm package was used for LQMM.

## Results

The baseline characteristics of patients are reported in Table 1. 56 patients were enrolled in this study (28 for the test group and 28 for the control group). No patients were lost during the follow-up and no adverse events were noted. 12 females and 16 males were recruited for the test group with a mean age of 24,6 ± 4,24 years. 13 females and 15 males were recruited for the control group with a mean age of 26,9 ± 7,26 years. 12 total impacted third molars and 16 partial impacted third molars were extracted in the test group. 13 total impacted third molars and 15 partial impacted third molars were extracted in the control group. Based on clinical judgment, no clinically important baseline differences were identified between groups.

**Table 1:** Baseline characteristics of patients

	Total (*N* = 56)	Control (*N* = 28)	i-PRF (*N* = 28)
Age			
Mean (SD)	25.7 (6.0)	26.9 (7.3)	24.6 (4.2)
Gender			
Male	31 (55.4%)	15 (53.6%)	16 (57.1%)
Female	25 (44.6%)	13 (46.4%)	12 (42.9%)
Type molar Inclusion			
Bone	25 (44.6%)	13 (46.4%)	12 (42.9%)
Osteomucosal	31 (55.4%)	15 (53.6%)	16 (57.1%)
Dur. Intervention			
Mean (SD)	43.0 (14.2)	45.8 (17.0)	40.2 (10.3)
Preoperative Trago-ogonion			
Mean (SD)	14.0 (1.0)	13.9 (1.1)	14.1 (0.9)
Preoperative Gonion-Commissure Labialis			
Mean (SD)	8.9 (0.9)	9.0 (1.0)	8.9 (0.9)
Total Swelling			
Mean (SD)	23.0 (1.7)	23.0 (1.9)	23.0 (1.6)

### Swelling

The results related to the total swelling are reported in Table 2 and Figure S1 (supple

mentary 1). The interaction term between group and time was found to be statistically significant (*p* < 0.001), indicating that the treatment effect varied over time. At baseline, total swelling was 11.5 ± 0.96 mm for control group and 11.5 ± 0.79 mm for test group. No statistically significant differences were found (*p* = 0.918). On day 3 the total swelling was 12.7 ± 0.92 mm for control group and 12.1 ± 0.75 mm for test group corresponding to a mean reduction of 0.6 mm. A statistically significant difference was found (*p* = 0.006). On day 7 the total swelling was 12.3 ± 0.88 mm for control group and 11.7 ± 0.73 mm for test group. A statistically significant difference was found (*p* = 0.018). Swelling increased significantly over time in both groups. These findings are illustrated in Supplementary Figure S1.

**Table 2:** Total swelling results

	Time					Main Effects (Group, Time, Interactions)		
	***Baseline***	***Day 3***	***Day 7***	***p-value***		***Group*** ***(p-value)***	***Time*** ***(p-value)***	Group*Time ***(p-value)***
Tot. Swelling				***b.vs.3***	***b. vs. 7***	/	/	*< 0.001*
Control	11.5 ± 0.96	12.7 ± 0.92	12.3 ± 0.88	*< 0.001*	*< 0.001*			
i-PRF	11.5 ± 0.79	12.1 ± 0.75	11.7 ± 0.73	*< 0.001*	0.007			
p-values	*0.918*	*>0.006*	*0.018*					

Data are presented as mean(± sd); p value adjustment: tukey method

The linear regression model used to evaluate the relation between duration of intervention and total swelling at day 3 showed that the interaction between treatment group and duration of intervention was not statistically significant (*p* = 0.185), indicating that the relationship between total swelling and duration of intervention does not differ across treatment groups. Furthermore, the regression coefficient for duration of intervention, which quantifies the relation with total swelling, was not statistically significant (β = 0.01, *p* = 0.276).

### Pain

The results related to pain are reported in Table 3 and Figure S2 (Supplementary 2). On day 1, VAS was 9 [8; 10] for control group and 8 [7; 8] for test group. On day 4 VAS was 6 [5; 6.6] for control group and 3 [2; 4] for test group. On day 7 VAS was 2 [0.75; 3] for control group and 0 [0; 1] for test group. The interaction term between group and time was not statistically significant (*p* = 0.443), indicating that the treatment effect did not vary over time. Pain significantly decreases over time (*p* < 0.001), and difference between i-PRF and control groups were statistically significant (*p* < 0.001). These findings are illustrated in Supplementary Figure S2.

**Table 3:** Linear quantile mixed effect analysis

Time						Main effects (Group, Time, Interactions)		
	***Day 1***	***Day 4***	***Day 7***	***p-value***		***Group*** ***(p-value)***	***Time*** ***(p-value)***	Group*Time ***(p-value)***
VAS				***b.vs.3***	***b. vs. 7***	*< 0.001*	*< 0.001*	0.443
Control	9[8; 10]	6[5; 6.6]	2[0.75; 3]	/	/			
i-PRF	8[7; 8]	3[2; 4]	0[0; 1]	/	/			
p-values	/	/	/					

Data are presented as median [Q_1_; Q_3_]

### Wound healing

The results related to the wound healing values are reported in Table 4 and Figure S3 (supplementary 3). The interaction term between group and time was found to be statistically significant (*p* = 0.009). indicating that the treatment effect varied over time. Healing Index scores were consistently higher in the i‑PRF group at all time points, indicating accelerated soft tissue maturation. On day 3 the median of wound healing was 3 [2; 3] for control group and 3 [3; 3.25] for test group. A statistically significant difference was found (*p* = 0.001). On day 7 the median of wound healing was 3,5 [3; 4] for control group and 4 [4; 4] for test group. A statistically significant difference was found (*p* = 0.019). On day 14, the median of wound healing was 4 [4; 5] for control group and 5 [4; 5] for test group. A statistically significant difference was found (*p* = 0.001). On day 21, the median of wound healing was 5 [4; 5] for control group and 5 [5; 5] for test group. A statistically significant difference was found (*p* = 0.026). These findings are illustrated in Supplementary Figure S3.

**Table 4:** Linear quantile mixed effect analysis

Time								Main Effects (Group, Time, Interactions)		
	***Day 3***	***Day 7***	***Day 14***	***Day 21***	***p-value***			***Group*** ***(p-value)***	***Time*** ***(p-value)***	Group*Time ***(p-value)***
Wound healing					***3 vs. 7***	*** 7 vs. 14***	*** 14 vs. 28***	/	/	0.009
Control	3[2; 3]	3.5[3; 4]	4[4; 5]	5[4; 5]	* < 0.001*	* < 0.001*	* 0.021*			
i-PRF	3[3; 3.25]	4[4; 4]	5[4; 5]	5[5; 5]	* < 0.001*	* < 0.001*	* 0.941*			
p-values	*0.001*	*0.019*	*0.001*	0.026						

## Discussion

This study was designed to evaluate the efficacy of i-PRF, in comparison to spontanoeus healing, on the control of swelling, pain, and wound healing following impacted mandibular third molar surgery. To date, there is limited evidence evaluating the effect of submucosal i‑PRF in third molar surgery. The results of this randomized controlled clinical trial suggest that submucosal infiltration of i-PRF significantly reduces early postoperative swelling and pain, and enhances surgical wound healing following lower third molar extractions.

Postoperative swelling is a normal physiological response to tissue injury, particularly following surgical procedures such as wisdom tooth extraction [32]. This swelling is primarily driven by tissue trauma and the subsequent inflammatory response, which includes increased vasodilation and capillary permeability. These vascular changes permit the leakage of plasma and immune cells into the surrounding tissues. Inflammatory mediators such as histamine, prostaglandins, and cytokines are released, contributing to the development of edema. As immune cells migrate to the affected area, fluid accumulates in the interstitial spaces, resulting in visible swelling [33]. Typically, swelling peaks around 48–72 h after surgery and gradually subsides as inflammation resolves and healing progresses [34].

Our findings demonstrate a significant interaction between treatment group and time, indicating that the reduction in postoperative swelling varied between the control and test groups over the healing period (*p* < 0.001). At baseline, both groups presented with comparable swelling measurements confirming similar clinical status prior to intervention. By postoperative day 3, the test group exhibited significantly less swelling than the control group (12.1 ± 0.75 mm vs. 12.7 ± 0.92 mm; *P* = 0.006), and this difference remained statistically significant on day 7 (11.7 ± 0.73 mm vs. 12.3 ± 0.88 mm; *P* = 0.018). While swelling increased in both groups following surgery, as expected, the test group showed a more controlled inflammatory response and a faster reduction in soft tissue edema. Although statistically significant, the reduction in swelling observed in the i‑PRF group was approximately 0.6 mm less than in the control group on postoperative days 3 and 7. From a patient-centered perspective, this modest volumetric difference is unlikely to produce a visibly appreciable change in facial appearance or substantially affect functional recovery. Nevertheless, even small reductions in edema may help decrease tension on wound margins, thereby supporting more favorable soft tissue healing and contributing to improved early postoperative comfort.

This improvement can be attributed to the angiogenic and anti-inflammatory effects of i-PRF, that, with its high concentration of autologous growth factors and leukocytes, can modulate the inflammatory cascade [35]. These bioactive molecules stimulate neovascularization and regulate the inflammatory response, accelerating the resolution of inflammation [36]. By attenuating the severity and duration of inflammation, promoting soft tissue regeneration, and minimizing interstitial fluid accumulation, i-PRF contributed to a measurable reduction in postoperative swelling [37].

Our findings are not in line with those reported in previous studies. In a study conducted by Barone et al., the use of PRF in the form of plugs and membranes during the surgical extraction of impacted mandibular third molars did not show a statistically significant effect on postoperative swelling at days 1, 3, or 7 compared to the control group [38]. Notably, our study employed a manual measurement for swelling assessment, which may differ in sensitivity and precision from 3D facial scanning method used by Barone et al. This variation in evaluation technique—digital versus manual—could potentially account for the discrepancies in outcomes observed between studies. Additionally, the different forms of PRF—such as plugs versus infiltration—may have varying effects on postoperative edema.

Although the mean surgical time did not differ significantly between groups, the control group procedures were on average 5 min longer. This difference, although not statistically significant, may have influenced postoperative inflammation and is acknowledged as a potential confounder. Moreover, our findings indicate that the correlation between total swelling and duration of intervention was not statistically significant (*p* = 0.314). This contrasts with previous studies that reported a positive association, where the extent of facial swelling increased with longer operating times [39, 40]. This discrepancy could be attributed to differences in surgical techniques, postoperative protocols, or the sensitivity of swelling measurement methods across studies. Additionally, variations in patient demographics, or methods of measuring swelling could contribute to the observed divergence. It is also plausible that the threshold beyond which operating time significantly affects swelling was not reached in our cohort. Further investigation with a standardized methodology and larger sample sizes may help clarify this relationship.

Similarly to swelling, pain scores were found consistently lower in the i-PRF group, with a statistically significant difference noted on postoperative days 1 and 3. This aligns with a growing body of literature supporting the analgesic benefits of platelet concentrates in oral surgery. Numerous studies have demonstrated that i-PRF likely exerts its analgesic effect through anti-inflammatory cytokines and neuroprotective pathways (e.g., IL-4, IL-10, TGF-β), which reduce nociceptive signaling and tissue irritation [41, 42]. Moreover, platelet concentrates have mild antimicrobial and antioxidant properties, which can reduce infection-related inflammation and associated pain [43, 44]. These consistent findings across various clinical trials reinforce the therapeutic potential of i-PRF in improving patient comfort following surgical procedures.

Wound healing was another parameter in which i-PRF demonstrated superior outcomes. From day 3 onward, the Healing Index scores were consistently higher in the test group, reflecting accelerated tissue maturation and epithelialization. This enhanced healing response may be attributed to the sustained release of bioactive proteins and growth factors from i-PRF, which likely support cellular proliferation, angiogenesis, and matrix remodeling throughout the 28-day follow-up period. Platelet-derived growth factor (PDGF), Transforming growth factor-beta (TGF-β), Vascular endothelial growth factor (VEGF) and Epidermal growth factor (EGF) are involved in cell proliferation and differentiation, angiogenesis and extracellular matrix formation [45, 46]. i-PRF promotes faster epithelial cell migration and proliferation, leading to quicker closure of wounds and fibroblast proliferation, essential for connective tissue repair, making it is effectiveness in various surgical contexts, as such as periodontal regeneration, sinus augmentation, and peri-implant soft tissue management [47–51].

While the results of this randomized controlled trial are promising, several limitations should be considered. The relatively small sample size, although adequate for detecting differences in primary outcomes, may limit the generalizability of the findings to broader populations. Additionally, the short follow-up period of 28 days does not allow for the evaluation of long-term outcomes such as bone regeneration or the sustained stability of soft tissue healing. Some of the outcome measures, including pain assessment through the visual analogue scale (VAS) and wound healing via the Landry Healing Index, are subjective and may be influenced by individual variability despite efforts to ensure consistency through blinded evaluations. The method used to measure facial swelling, which relied on manual measurements with a flexible ruler, may lack the precision of more advanced technologies like three-dimensional facial scanning or ultrasound, potentially introducing measurement bias. Furthermore, the study was conducted in a single center, which could limit the applicability of the findings to different clinical settings with varying surgical techniques or patient populations. Although the co-investigator responsible for measurements and statistician remained unaware of the specific procedure applied to each patient, the principal investigator was not blinded due to the experimental procedures. Another limitation of this study is the absence of a placebo (e.g., saline) injection in the control group. While both groups received identical surgical and postoperative care, the knowledge that an injection was administered in the test group may have introduced a perception bias, particularly for patient-reported outcomes such as pain intensity and subjective comfort. Patients receiving an additional intervention might have perceived greater care or anticipated better outcomes, which could partially influence their pain reporting and overall recovery perception. Future studies should consider a placebo-controlled design, where the control group receives a placebo injection, to better isolate the true biological effects of i‑PRF from potential psychological or placebo-related influences.

 Moreover, in our protocol, ibuprofen 400 mg was prescribed only on demand, with a maximum of two doses permitted during the follow-up period; patients exceeding this limit were excluded. Although this strategy was intended to standardize postoperative management, we acknowledge that on-demand intake may still introduce variability in pain perception and swelling, as analgesic use was not strictly controlled across all patients. Furthermore, the limited frequency of intake prevented meaningful subgroup analyses. Future trials should adopt stricter monitoring of postoperative medication use, or consider standardized rescue protocols, to reduce this potential source of bias. Another important methodological consideration is the use of a parallel-group design rather than a split-mouth approach. While split-mouth trials are often preferred in oral surgery research due to their ability to minimize inter-individual variability and increase statistical power, we opted for a parallel design based on both clinical and methodological considerations. Many patients presented with only one impacted lower third molar or with asymmetrical extraction difficulty, which would have compromised internal consistency in a split-mouth setting. To minimize variability, we applied strict inclusion criteria and ensured group comparability in terms of age, sex, baseline swelling, and impaction characteristics. Nevertheless, the absence of a split-mouth design is acknowledged as a limitation, and future studies employing this model may offer additional insights into the localized efficacy of i-PRF.

Future studies should incorporate objective and standardized measurement tools, such as three-dimensional (3D) facial scanning or ultrasonography, which allow for volumetric analysis and provide higher reproducibility and sensitivity to subtle changes in facial edema.

## Conclusions

Within the limit of our study including its relatively small sample size, single-center setting, on-demand use of postoperative ibuprofen and the absence of a split-mouth design, this randomized controlled clinical trial suggests that submucosal infiltration of injectable platelet-rich fibrin (i-PRF) after the extraction of impacted lower third molars may help reduce postoperative swelling and pain, while also promoting faster wound healing. These findings support the potential of i-PRF as a safe and minimally invasive adjunct in oral surgical procedures, to enhance patient recovery and reduce early complications. particularly in third molar extractions. Although the observed differences were statistically significant, the clinical relevance of the swelling reduction remains to be established. Further investigations with a standardized methodology and larger sample sizes may help clarify this effect and assess long-term benefits.

## Supplementary Information

Below is the link to the electronic supplementary material.

Supplementary Material 1 (DOCX 177 KB)

## Data Availability

Data is provided within the manuscript or supplementary information files.
