# Supplementary material for: Using injectable Platelet-Rich fibrin to improve recovery after impacted lower third molar extraction: a randomized controlled clinical trial
Source: Clin Oral Investig. 2025 Sep 19;29(10):467. doi: 10.1007/s00784-025-06563-3 (PMC12449355; doi:10.1007/s00784-025-06563-3)
Supplement: Supplementary file 1 — Supplementary Material 1 (DOCX 177 KB) [file 784_2025_6563_MOESM1_ESM.docx]

**
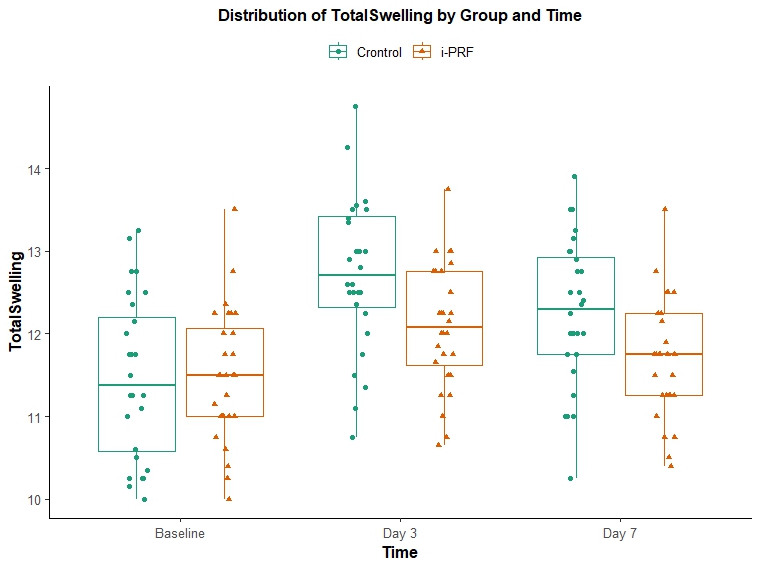
**

Figure S1: Boxplot of the distribution of Total Swelling by Group and Time


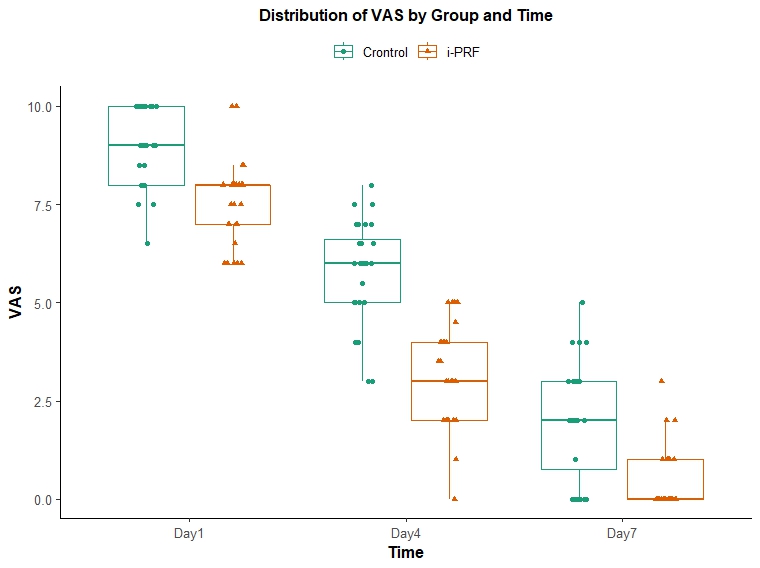


Figure S2: Boxplot of the distribution of Total Swelling by Group and Time

**
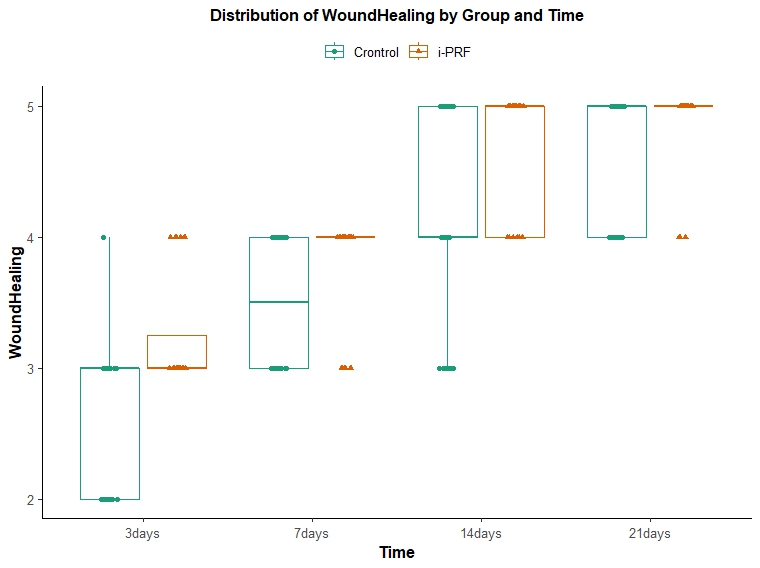
**

Figure S3: Boxplot of the Distribution of Wound Healing by Group and Time
